# Characterization of DNase activity and gene in *Streptococcus suis* and evidence for a role as virulence factor

**DOI:** 10.1186/1756-0500-7-424

**Published:** 2014-07-04

**Authors:** Bruno Haas, Laetitia Bonifait, Katy Vaillancourt, Steve J Charette, Marcelo Gottschalk, Daniel Grenier

**Affiliations:** 1 Groupe de Recherche en Écologie Buccale (GREB), Faculté de Médecine Dentaire, Université Laval, 2420 Rue de la Terrasse, Quebec City, Quebec G1V 0A6, Canada; 2 Institut de Biologie Intégrative et des Systèmes (IBIS), Quebec City, Quebec, Canada; 3 Centre de Recherche de l’Institut Universitaire de Cardiologie et de Pneumologie de Québec (CRIUCPQ), Hôpital Laval, Quebec City, Quebec, Canada; 4 Département de Biochimie, de Microbiologie et de Bio-Informatique, Faculté des Sciences et de Génie, Université Laval, Quebec City, Quebec, Canada; 5 Centre de Recherche en Infectiologie Porcine et Avicole (CRIPA), Fonds de Recherche du Québec - Nature et Technologies (FRQNT), Saint-Hyacinthe, Quebec, Canada; 6 Groupe de Recherche sur les Maladies Infectieuses du Porc (GREMIP), Faculté de Médecine Vétérinaire, Université de Montréal, Saint-Hyacinthe, Quebec, Canada

**Keywords:** *Streptococcus suis*, nuclease, DNase activity, Virulence factor, SsnA

## Abstract

**Background:**

The Gram-positive bacterium *Streptococcus suis* serotype 2 is an important swine pathogen and emerging zoonotic agent. Multilocus sequence typing allowed dividing *S. suis* serotype 2 into sequence types (STs). The three major STs of *S. suis* serotype 2 from North America are 1 (most virulent), 25 (intermediate virulence) and 28 (less virulent). Although the presence of DNase activity in *S. suis* has been previously reported, little data is available. The aim of this study was to investigate DNase activity in *S. suis* according to STs, to characterize the activity and gene, and to provide evidence for a potential role in virulence.

**Results:**

We showed that ST1 and ST28 strains exhibited DNase activity that was absent in ST25 strains. The lack of activity in ST25 isolates was associated with a 14-bp deletion resulting in a shifted reading frame and a premature stop codon. The DNase of *S. suis* P1/7 (ST1) was cell-associated and active on linear DNA. A DNase-deficient mutant of *S. suis* P1/7 was found to be less virulent in an amoeba model. Stimulation of macrophages with the DNase mutant showed a decreased secretion of pro-inflammatory cytokines and matrix metalloproteinase-9 compared to the parental strain.

**Conclusions:**

This study further expands our knowledge of *S. suis* DNase and its potential role in virulence.

## Background

*Streptococcus suis* is an important swine pathogen worldwide that causes mainly meningitis, arthritis, endocarditis, and septicemia [1]. It can also affect humans in close contact with infected pigs or contaminated pork-derived products [2,3]. More particularly, two major *S. suis* outbreaks with symptoms of toxic shock-like syndrome occurred in 1998 and 2005 in China and caused over 50 deaths [4]. To date, thirty-five serotypes have been described and serotype 2 is the most commonly isolated from diseased pigs and humans [5]. In addition, *S. suis* is classified into numerous sequence types (STs) by multilocus sequence typing (MLST) [6]. A recent study showed that most isolates of *S. suis* serotype 2 from North America are part of three major STs: ST1, ST25 and ST28 showing high, intermediate and low virulence in a mouse model, respectively [7]. Despite several virulence factors already identified, the pathogenesis of *S. suis* infections is not fully understood, especially regarding the mechanisms that allow the bacterium to escape from the host immune system [8]. The best characterized factor providing resistance of *S. suis* to the host immune system is the sialic acid-rich capsule [8]. More specifically, the capsule confers resistance to phagocytosis by macrophages, and mutants deficient for capsule expression are less virulent in mouse and pig models of infection [9,10]. Moreover, unencapsulated mutants are also more sensitive to antibiotics, including penicillin G and ampicillin [11].

Evidence has been brought to support a role of bacterial deoxyribonucleases (DNases), which are enzymes that hydrolyze nucleic acids to yield oligonucleotides, as virulence factors. More specifically, DNases may be involved in bacterial growth [12] and biofilm maturation [13], as well as in the ability of bacteria to escape the immune system [14]. In 2004, a cell-associated DNase (112 kDa), encoded by the *ssnA* gene, has been identified in *S. suis* serotype 2 [15]. The recent pan-surfome analysis performed on *S. suis* and which concluded that the cell-associated DNase may represent one of the best vaccine candidates has revived the interest for this protein [16]. More specifically, it was found that the *S. suis* DNase is largely distributed among serotypes, and is highly immunogenic and accessible to antibodies [16]. In addition, de Buhr et al. [17] recently reported that *S. suis* DNase is involved in the degradation and escaping of neutrophil extracellular traps. The aim of the present study was to investigate the distribution of DNase activity among the three major STs (ST1, ST25, and ST28) of *S. suis*, to characterize the activity and gene, and to provide evidence for a potential role in the pathogenic process of *S. suis* infections.

## Methods

### Bacterial strains and culture conditions

Strains of *S. suis* used in this study and their origin are listed in Table 1. Bacteria were routinely grown in Todd-Hewitt Broth (THB) (BBL Microbiology Systems, Cokeysville, MD, USA) at 37°C.

**Table 1:** **Sequence type (ST), origin, and DNase activity of *S. suis *** **serotype 2 strains used in this study**

**Strain**	**ST**	**Origin**	**DNase activity**^**1**^
P1/7	1	Europe	+
M2D^2^	NA^3^	NA	-
MNCM01	1	Thailand	+
MNCM06	1	Thailand	+
MNCM16	1	Thailand	+
MGGUS2	1	USA	+
MGGUS3	1	USA	+
NIAH11433	1	Japan	+
DAT261	1	Japan	+
DAT264	1	Japan	+
DAT229	1	Japan	+
1043248	25	Canada	-
1043629	25	Canada	-
1044423	25	Canada	-
1053253	25	Canada	-
1085543	25	Canada	-
MNCM04	25	Thailand	-
MNCM10	25	Thailand	-
MNCM24	25	Thailand	-
MNCM26	25	Thailand	-
MNCM51	25	Thailand	-
MGGUS4	25	USA	-
MGGUS5	25	USA	-
89/1591	25	Canada	-
1054471	28	Canada	+
1088563	28	Canada	+
1097205	28	Canada	+
1057906	28	Canada	+
MNCM43	28	Thailand	+
MGGUS9	28	USA	+
MGGUS10	28	USA	+
MGGUS11	28	USA	+
MGGUS12	28	USA	+
DAT292	28	Japan	+
DAT242	28	Japan	+
DAT245	28	Japan	+
DAT246	28	Japan	+

^1^Determined with the DNase Test Agar medium. (+), presence of a clear zone around bacterial growth; (-), absence of a clear zone.

^2^DNase-deficient mutant from *S. suis* P1/7.

^3^Not applicable.

### Determination of DNase activity

Strains of *S. suis* were screened for DNase activity using a plate assay. Briefly, 50 μl of overnight broth cultures of *S. suis* were spotted on the surface of DNase Test Agar plates (BBL Microbiology Systems) which were incubated for 24 h at 37°C. After adding 0.1 N HCl for 5 min, the appearance of a clear zone around bacterial growth indicated degradation of DNA. DNase activity of *S. suis* P1/7 was also determined and characterized by quantification of residual DNA after incubation of bacterial cells or filtered culture supernatants with DNA using the Quant-iT™ PicoGreen® dsDNA Reagent (Invitrogen, Eugene, OR, USA) according to the manufacturer’s protocol, as previously described [18], with some modifications. Briefly, linear double-stranded salmon sperm DNA (Sigma-Aldrich Canada Co., Oakville, ON, Canada) was prepared in DNA buffer (5.96 g/l HEPES [N-2-hydroxyethylpiperazine-N’-2-ethanesulfonic acid], 590 mg/l CaCl_2_.2H_2_O, and 380 mg/l MgCl_2_, pH 7.5 prepared in RNase/DNase-free water) at 100 μg/ml, and incubated with cells of *S. suis* (OD_660_ = 1 in DNA buffer) or filtered culture supernatants. To determine heat stability of DNase activity, cells of *S. suis* were preheated for 30 min at 50, 60 or 70°C prior to adding double-stranded salmon sperm DNA in the assay. A range of pH between 6 and 8 with increment of 0.5 was used to determine the optimal pH for DNase activity. Ion chelators (ethylenediaminetetraacetic acid [EDTA], ethylene glycol tetraacetic acid [EGTA] and 1,10-phenanthroline) were tested for inhibition of *S. suis* DNase activity at 1 and 10 mM. Following incubation (3 h) at 37°C (or 25, 30 and 42°C for optimal temperature determination), samples were centrifuged 5 min at 11,000 g and residual DNA was quantified by adding the PicoGreen® reagent. After 5 min at room temperature, fluorescence was measured with a Synergy 2 BioTek microplate reader (BioTek Instruments, Inc, Winooski, VT, USA) using excitation wavelength of 485 nm and an emission wavelength of 528 nm.

### Activity spectrum of *S. suis* DNase activity

*S. suis* P1/7 cells from an overnight culture were harvested by centrifugation and suspended in 10 mM Tris–HCl buffer (pH 7.5) containing 5 mM CaCl_2_ and 4 mM MgCl_2_ to an OD_660_ = 1. Cells were incubated with 40 ng/μl (final concentration) linear DNA (lambda DNA [Promega, Madison, WI, USA]) or 32.5 ng/μl (final concentration) circular plasmid DNA (pUC18) for 3 h at 37°C and degradation was monitored following migration in a 0.8% agarose gel (45 min, 110 V) and staining with EZ-Vision™ Three (Amresco, Solon, OH, USA).

### Genotyping and sequence analysis

Three strains of *S. suis* belonging to ST1 (P1/7, MNCM01, MGGUS2), ST25 (1043248, MNCM04, MGGUS4) and ST28 (1097205, MNCM43, MGGUS9) were grown for 24 h on THB agar plates. Colonies were suspended in sterile water, boiled for 10 min and centrifuged at 10,000 g for 3 min. PCR was then performed using the following primers in a reaction mixture containing 5 U EconoTaq® (Lucigen, Middleton, WI) and 1.5 mM MgCl_2_: ssnA 3088 F (5′ GAC GTC CAT ATA TAA CAA AAA GGA G 3′) and ssnA 5392R (5′ GTC GAT TCG GCC TAG GCT GAG ATT G 3′) or ssnA 3884 F (5′ ATT ACA GAA ACA AAC ATC GCT CAG T 3′) and ssnA 6278R (5′ ACA AGT GGA GGT GGA GCA GTA GAA A 3′). Thirty PCR cycles were performed starting with 1 min denaturation at 94°C, 1 min hybridization at 56°C and 3 min elongation at 72°C. Products were sequenced and compared to the *ssnA* sequence of *S. suis* P1/7 (ST1) using BioEdit and the NCBI database. BLASTp was used to compare the predicted amino acid sequence of the DNase with that of similar proteins identified in other streptococcal species.

### Isolation and characterization of a DNase deficient mutant

A mutant library constructed in a previous study [19] by using the pTV408 temperature sensitive suicide vector to deliver the Tn*917* transposon into *S. suis* P1/7 *via* electroporation was screened with the DNase plate assay to recover a deficient mutant. The number of transposon Tn*917* insertion in the DNase deficient mutant (M2D) was determined by southern blot using a digoxygenin (DIG)-labeled probe specific to the *erm* gene of transposon Tn*917* as previously described [20]. The exact site of insertion of transposon Tn*917* in mutant M2D was determined using plasmid rescue. Briefly, genomic DNA was extracted and digested using HindIII, ligated and PCR was then performed on the ligature mix using the primers Erm-F (5′ ACG AGT GAA AAA GTA CTC AAC C 3′) and Tn*917* (5′ AGA GAG ATG TCA CCG TCA AGT 3′), 5 U EconoTaq® (Lucigen) in 1.5 mM MgCl_2_. Thirty PCR cycles were performed starting with 1 min denaturation at 94°C followed by 1 min hybridization at 50°C and 5 min elongation at 72°C. Amplicons were then sequenced (Genomic Analysis Platform of Université Laval, Québec, QC, Canada).

### Virulence assay in an amoeba model

The DNase-deficient mutant (M2D) and its parental strain (P1/7) were tested using the amoeba host model *Dictyostelium discoideum*. In a previous study [21], we showed that *D. discoideum* cannot multiply and form plaques on lawns of virulent strains of *S. suis*, including P1/7, whereas plaque formation was observed on lawns of mutants (capsule, subtilisin-like protease) of *S. suis* known to be avirulent in an animal model. Briefly, amoebae were grown in HL5 liquid medium (14.3 g/l peptone, 7.15 g/l yeast extract, 18 g/l maltose, 3.6 mM Na_2_HPO_4_, 3.6 mM KH_2_PO_4_) containing tetracycline (15 μg/ml). Amoebae were harvested by centrifugation (5 min at 1,500 g), washed, and resuspended in tetracycline-free HL5 medium at a concentration of 300, 150, 75, 38, 18, or 9 amoeba cells per 5 μl. Meanwhile, bacteria were harvested by scraping the surface of two Petri plates with confluent lawns of growth (overnight at 37°C) and suspended in 2 ml of HL5 medium without tetracycline. The bacterial suspensions (100 μl, OD_660_ ≈ 5) were then applied to wells of 24-well plates containing HL5 agar medium (2 ml/well). Once dried, bacterial lawns were spotted with 5 μl of the *D. discoideum* suspensions. Plates were incubated at 23°C for 2 days and then examined visually for plaque formation. Three independent experiments were performed to ensure reproducibility.

### Cytokine and MMP-9 secretion by a macrophage model

The human monoblastic leukemia cell line U937 (ATCC CRL-1593.2; American Type Culture Collection, Manassas, VA, USA) was cultivated at 37°C in a 5% CO_2_ atmosphere in RPMI-1640 medium (HyClone Laboratories, Logan, UT, USA) supplemented with 10% heat-inactivated fetal bovine serum (FBS; RPMI-FBS) and 100 μg/ml penicillin-streptomycin. Differentiation into macrophage-like cells with phorbol 12-myristate 13-acetate was performed as previously described [22]. One million macrophage-like cells were seeded in 12-wells culture plates and incubated overnight at 37°C in 5% CO_2_. Culture medium was then aspirated and replaced with fresh RPMI-1640 supplemented with 1% heat-inactivated FBS and 100 μg/ml penicillin-streptomycin containing *S. suis* (DNase-deficient mutant [M2D] or parental strain [P1/7]) at multiplicity of infection (MOI) of 100, 50 or 10. After 24 h of stimulation at 37°C in 5% CO_2_, supernatants were collected. Quantification of interleukin-6 (IL-6), interleukin-8 (CXCL8), tumor necrosis factor-α (TNF-α), and matrix metalloproteinase 9 (MMP-9) was performed by enzyme-linked immunosorbent assays (ELISA) (eBioscience Inc., San Diego, CA, USA) following the manufacturer’s instructions. All treatments were performed in triplicate. Differences between means were analyzed for statistical significance using the Student’s t-test and were considered significant at *p* < 0.05.

## Results

### Distribution of DNase activity and comparative analysis of *ssnA* gene in *S. suis* according to STs

**Figure 1:**
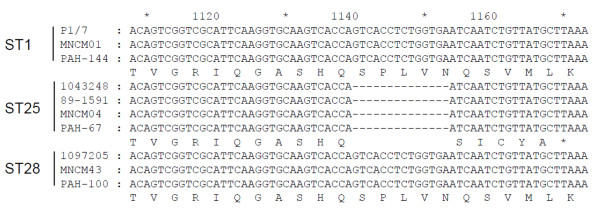
**Comparative analysis of the *ssnA *gene according to STs.** Nucleic acid and predicted amino acid sequences of *ssnA* gene (from position 1108 to 1173) of *S. suis* belonging to ST1, ST25, and ST28 were compared in order to identify the origin of the loss of DNase activity in ST25.

Thirty-six *S. suis* serotype 2 strains were tested for DNase activity using the plate assay. All strains grew similarly on the DNase Test Agar medium. As reported in Table 1, strains belonging to ST1 and ST28 all possessed DNase activity whereas ST25 strains did not show any capacity to degrade DNA. A comparative analysis of the *ssnA* gene of *S. suis* strains belonging to either ST1, ST25 or ST28 was performed. Sequence comparison of the *ssnA* gene of *S. suis* P1/7 (ST1; *SSU1760*; UniProt accession no. C5VVJ6) with those amplified from *S. suis* serotype 2 belonging to each of the three STs showed that the nucleic acid sequence of *ssnA* gene of *S. suis* P1/7 is identical to that of corresponding genes found in *S. suis* isolates belonging to ST1 group. The *ssnA* gene from P1/7 showed a % identity of 98.1% and 98% with corresponding genes in strains of the ST25 and ST28 groups, respectively. However, when the predicted amino acid sequences were analyzed, the ST25 group (devoid of DNase activity) showed only 36.3% identity with the predicted protein of gene *SSU1760* of *S. suis* P1/7 whereas ST28 and ST1 groups showed 98.4% and 100% identity, respectively. A detailed sequence analysis revealed that the low % identity in the predicted protein of *S. suis ssnA* gene of the ST25 group is related to a truncated form of the protein due to the loss of 14 bp region starting from position 1140 (Figure 1). A shifted reading frame is associated with this deletion, generating a stop codon starting at position 1170.

### Genetic organization of *ssnA* gene (SSU1760)

The *ssnA* gene is preceded by *gshA* (glutamate cysteine ligase/glutathione synthetase) and followed by *hslO* (Hsp33-like chaperonin). This genetic organization is conserved in all published genomes of *S. suis* (data not shown). The open reading frame (ORF) of *S. suis* P1/7 *ssnA* is 3120 bp in length and codes for a 1039 amino acid protein. The resulting SsnA protein possesses the Gram positive cell wall anchoring motif (LPKTG) at the C-terminus end followed by a hydrophobic domain, while a signal peptide is found at the N-terminus end. It also has two oligonucleotide/oligosaccharide-binding (OB)-fold domains. The active site belongs to the endonuclease/exonuclease/phosphatase family domain according to Kegg database (http://www.genome.jp/dbget-bin/www_bget?ssi:SSU1760).

### Percentage identity of the DNase SsnA (SSU1760) with similar proteins found in pathogenic streptococci

Comparison of the predicted amino acid sequence of DNase SsnA (SSU1760) with similar proteins found in other pathogenic streptococcal species showed that the protein shares 48.7% and 48.4% identity with SpnA and spyM18_0808, respectively, which are both cell-wall anchored DNases of *Streptococcus pyogenes*. The predicted nucleic acid-binding domain (IT**E**TN**I**AQ**L**A**T**QAQA**TLV**S**L**KN) identified by Fontaine et al. [15] in the DNase contains several residues found in the *S. pyogenes* SpnA nucleic acid-binding domain (VK**E**AV**I**SE**L**E**T**TTPS**TLV**K**L**SH).

### Characterization of *S. suis* P1/7 DNase activity

**Figure 2:**
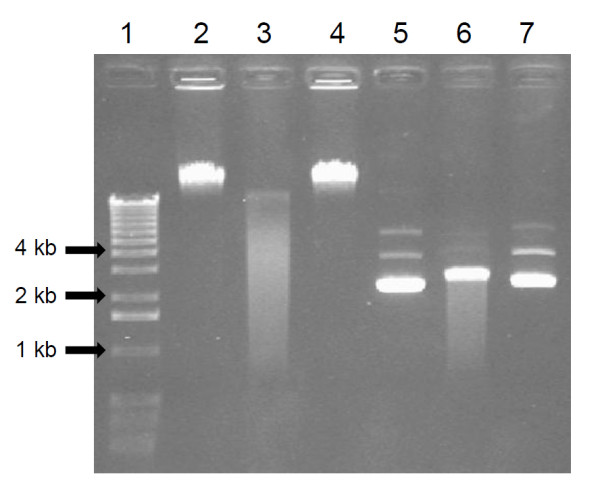
**Electrophoretic analysis of DNA degradation by *S. suis *P1/7 cells.** Lanes 1: DNA markers, 2: Control linear DNA (lambda DNA), 3: Linear DNA + *S. suis* P1/7, 4: Linear DNA + *S. suis* DNase deficient mutant (M2D), 5: Control plasmid DNA, 6: Plasmid DNA + *S. suis* P1/7, 7: Plasmid DNA + *S. suis* DNase deficient mutant (M2D).

The fluorogenic assay was used to determine the localization, stability and optimal conditions of DNase activity of *S. suis* P1/7 (Table 2). The activity was found to be cell-associated and not secreted in the culture supernatant. *S. suis* DNase activity was heat sensitive since less than 2% activity remained after treatment of bacteria at 50°C (30 min). The optimal pH for the activity ranged between pH 7 and 7.5. DNase activity was slightly stronger at a temperature of 42°C than 37°C. The activity was completely inhibited by EDTA (10 mM) and EGTA (10 and 1 mM) and partially inhibited by 1,10-phenanthroline. To determine the substrate specificity of *S. suis* DNase, linear DNA or circular plasmid DNA was incubated with bacterial cells prior to monitor degradation by electrophoresis. As shown in Figure 2, the band corresponding to linear DNA was degraded after incubation with *S. suis* P1/7 compared to the control without bacteria (compare lane 3 with lane 2). The migration profile of circular DNA alone (lane 5) showed 3 distinct bands, which correspond to nicked DNA (6 kb band), linear DNA (4 kb band) and supercoiled DNA (2.5 kb band) [23]. Incubation of circular DNA with the bacteria (lane 6) caused the appearance of a new band at 2.6 kb compared to the control sample. This band could correspond to supercoiled DNA bound to the DNase [23]. A decreased intensity of the two bands (4 kb and 6 kb) corresponding to the linear and nicked forms of the plasmid was observed.

**Table 2:** **Sublocalization, stability, optimal conditions and inhibition of DNase activity of *S.suis *** **P1/7**

**Treatment/condition**	**Activity (%) ± SD**
Sublocalization	
Pellets	100 ± 0.2
Supernatant	7.2 ± 1.0
Stability	
No treatment	100 ± 0.2
50°C/30 min	1.8 ± 0.3
60°C/30 min	1.9 ± 1.0
70°C/30 min	0 ± 1.8
Reactional pH	
6.0	38.5 ± 0.8
6.5	82.1 ± 0.5
7.0	100 ± 0.2
7.5	98.7 ± 0.1
8.0	96.4 ± 0.2
Incubation temperature	
25°C	10.0 ± 0.2
30°C	49.3 ± 0.3
37°C	100 ± 0.1
42°C	110.6 ± 0.05
Inhibition	
None	100 ± 13.0
EDTA 10 mM	0 ± 7.1
EDTA 1 mM	46.8 ± 9.7
EGTA 10 mM	0 ± 10.9
EGTA 1 mM	0 ± 10.2
1,10-phenanthroline 10 mM	33.3 ± 29.2
1,10-phenanthroline 1 mM	52.4 ± 1.9

### Isolation of a DNase-deficient mutant from a bank of mutants

A bank of mutants (1,150) of *S. suis* P1/7 prepared in a previous study [19] by insertion of transposon Tn*917* was screened using the DNase Test Agar medium, and one mutant (M2D) completely devoid of DNase activity was identified. The loss of DNase activity was also confirmed after incubation of linear DNA with the mutant M2D (Figure 2, lane 4). Southern blot analysis of genomic DNA was performed in order to determine the number of insertion in mutant M2D. A single band was identified supporting the presence of one insertion of transposon Tn*917* (data not shown). Using a plasmid rescue procedure, the insertion causing the loss of DNase activity was found to be in position 762 in *ssnA* gene.

### Virulence of the DNase-deficient mutant in an amoeba virulence model

The virulence assay using the amoeba model *D. discoideum* was used to compare *S. suis* P1/7 with its DNase-deficient mutant M2D. As reported in Table 3, ≥ 300 amoebae per well were needed to cause plaque formation on bacterial lawns of the parental strain. However, amoebae in the range of 9 to 18 cells per well were sufficient to induce plaque formation on lawns of the DNase-deficient mutant, thus indicating a much higher susceptibility to predation by *D. discoideum*.

**Table 3:** **Susceptibility of *S. suis *** **P1/7 and its DNase-deficient mutant (M2D) to predation of *D. discoideum***

**Strain**	**DNase activity**	**Virulence assay**		
		**Expt 1**	**Expt 2**	**Expt 3**
P1/7	+	>300	>300	300
M2D	-	18	9	9

### Inflammatory response induced by the DNase-deficient mutant in a macrophage model

**Figure 3:**
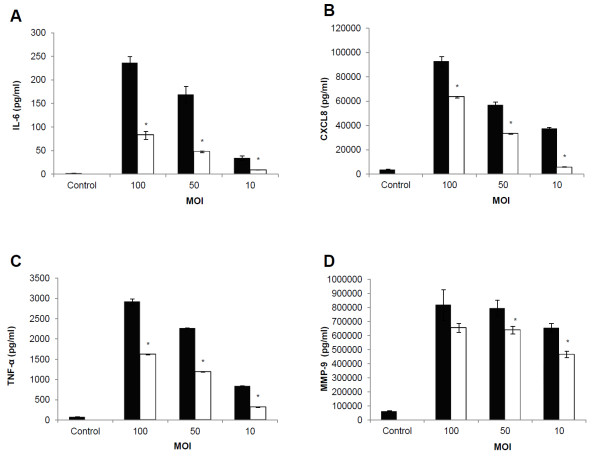
**Quantification of pro-inflammatory cytokines and MMP-9 produced by macrophages stimulated with *S. suis *P1/7 and M2D.** Secretion of IL-6 **(panel A)**, CXCL8 **(panel B)**, TNF-α **(panel C)** and MMP-9 **(panel D)** by macrophages stimulated with cells of *S. suis* P1/7 and its DNase-deficient mutant M2D at MOIs of 10, 50, and 100. *: *p* < 0.05. ■ *S. suis* P1/7, □ *S. suis* M2D.

The impact of the loss of DNase activity in the inflammatory response of *S. suis* cells in a macrophage model was investigated. Both *S. suis* P1/7 and its DNase-deficient mutant M2D induced IL-6, CXCL8, TNF-α and MMP-9 secretion by macrophages in a dose-dependent fashion (Figure 3). However, macrophages stimulated with cells of mutant M2D secreted significantly lower amounts of cytokines and MMP-9 in comparison with the parental strain. More specifically, at MOI of 100, the cells stimulated with mutant M2D produced 65.2%, 31.6%, 44.7% and 19.8% less IL-6, CXCL8, TNF-α and MMP-9 respectively, than the parental strain P1/7.

## Discussion

*S. suis* serotype 2 is responsible for important economical losses in the swine industry worldwide. In order to better understand the pathogenic process of *S. suis* infections, it is essential to identify all virulence factors produced by this pathogen and to evaluate their exact contribution in pathogenesis. In a previous study, Fontaine *et al.*[15] identified a cell wall-anchored DNase in *S. suis*. Given that DNases have been suggested as an important virulence factor in human pathogenic streptococci [24,25] and that very few is known on the DNase of *S. suis*, this study is of relevance. We first investigated the presence of this activity in the three major North American STs, namely ST1, ST25 and ST28 that differ in their virulence in a mouse model [7]. This comparative analysis suggests that a direct link between virulence in mice and the presence of DNase activity cannot be established. While strains belonging to the most virulent ST (ST1) and to the less virulent ST (ST28) were all positive for DNase activity, ST25 strains having an intermediate virulence lacked the activity. The absence of DNase activity in ST25 strains is explained by a 14-bp deletion from position 1140 to 1153 in *ssnA* gene of these strains causing a shifted reading frame that generates a stop codon starting at position 1170.

Considering that the insertion of a single transposon in mutant M2D resulted in a complete loss of DNase activity, it can be suggested that the DNase encoded by the *ssnA* gene is the only active extracellular DNase expressed by *S. suis* in the tested conditions. Interestingly, twenty-five genes coding for putative nucleases, including fourteen DNases are present in *S. suis* P1/7 genome (data not shown). Ones should not exclude that additional DNases strictly located in the cytoplasm and contributing to DNA catabolism and/or reparation can be expressed in addition to other extracellular DNase(s) only under specific conditions. No data are available on the localization of these putative nucleases.

The ability of *S. suis* DNase to degrade linear and not circular DNA, as stated previously [15], suggests that it has uniquely an exonuclease activity. The fact that circular DNA treated with *S. suis* P1/7 showed a decreased intensity of the bands corresponding to the nicked and linear forms of the plasmid could be the result of its partial degradation. The remaining band migrated at 2.6 kb rather than 2.5 kb in the control sample could correspond to the binding of the DNase to supercoiled DNA [23]. As opposed to the findings of Fontaine *et al.*[15], we found that *S. suis* P1/7 DNase was heat-sensitive. This discrepancy may be related to the fact that we used a more sensitive assay procedure to determine DNase activity. *S. suis* DNase was active at physiological pH and temperature, with an increased activity at 42°C suggesting that, in case of infection, fever might promote DNase activity. Inhibition of the activity by ion chelators such as EDTA, EGTA or 1,10-phenanthroline is in agreement with the fact that the DNase activity of *S. suis* depends on the presence of Ca^2+^ and Mg^2+^[15].

*D. discoideum* amoeba has been shown to be a suitable alternative host model to analyze the virulence of various pathogenic bacteria mainly due to its phagocytic activity and its easiness to use [26,27], prior to use an animal model. In a previous study, we showed that *S. suis* mutants deficient for capsule expression or for the production of the subtilisin protease were sensitive to amoeba predation suggesting a decreased virulence as the one seen in an animal model [21]. In this study, the DNase-deficient mutant was found to be more susceptible to amoeba predation compared to its parental strain. This suggests that DNase activity likely contributes to the virulence of *S. suis*, probably through an action on the phagocytic cells. Therefore, this decreased virulence should be confirmed in an animal model such as mouse or piglet.

To determine a potential contribution of *S. suis* DNase in the host inflammatory response, macrophages were stimulated with the DNase-deficient mutant and its parental strain and cytokine and MMP-9 secretion was monitored. It was found that macrophages stimulated with *S. suis* P1/7 secreted higher amounts of IL-6, CXCL8, TNF-α, and MMP-9 than those stimulated with M2D. To the best of our knowledge, this is the first report on the ability of a bacterial DNase to exert a pro-inflammatory effect on macrophages. This contribution of *S. suis* DNase in cytokine and MMP-9 secretion may be of utmost importance in the pathogenic process of meningitis. Indeed, Lopes-Cortes *et al.*[28] reported that TNF-α is present in the cerebrospinal fluid and that high levels of this cytokine correlate with neurological complications. Moreover, high concentrations of MMP-9 are observed *in vivo* in the cerebrospinal fluid during bacterial meningitis and in an experimental model of meningitis [29,30]. These results in addition to the one from the amoeba virulence assay are evidences that the *S. suis* DNase activity directly affects phagocytic cells confronted to the bacterium. DNases have often been described as virulence factors in streptococci [31] or staphylococci [14]. Indeed, it has been shown that DNase can help bacteria to escape from neutrophil extracellular traps (NETs) which are structures secreted by neutrophils to trap and kill bacteria [32]. These structures are mainly made of DNA, proteases, anti-microbial peptides and histones [33]. A recent study showed that *S. suis* DNase can degrade NETs to escape from the host innate immune system [17].

## Conclusions

In this study, we further characterized at the genetic and functional levels the *S. suis* cell wall-anchored DNase. We showed that this activity was present in all virulent strains belonging to ST1 and that less virulent isolates (ST25) lacked the activity. The absence of DNase activity was due to a conserved 14-bp deletion in the *ssnA* gene causing a shifted reading frame and apparition of an early stop codon resulting in a truncated and inactive protein. We showed that the DNase is active at physiological temperature and pH on linear DNA, and also brought evidences that it represents the most active DNase of *S. suis* since its inactivation completely abolished *S. suis* DNase activity. Furthermore, the DNase may contribute to the virulence of *S. suis* by increasing the inflammatory response.

## Competing interests

The authors declare that there is no competing interests regarding the publication of this article.

## Authors’ contributions

BH was the main experimentator for this study while LB carried out the screening of the *S. suis* P1/7 mutant library, isolated the DNase-deficient mutant and performed part of the virulence assays. KV was in charge of the molecular biology assays and analysis. SJC supervised the virulence assay. MG provided the *S. suis* strains. DG supervised and conceived this study. BH prepared the manuscript under the supervision of DG. All authors read and approved the manuscript.
